# Long‐term molecular remission in a patient with acute myeloid leukemia harboring a new *NUP98‐LEDGF* rearrangement

**DOI:** 10.1002/cam4.2051

**Published:** 2019-03-07

**Authors:** Maria Pilar Gallego Hernanz, José Miguel Torregrosa Diaz, Nathalie Sorel, Arthur Bobin, Elodie Dindinaud, Sabrina Bouyer, Deborah Desmier, Françoise Brizard, Xavier Leleu, Natacha Maillard, Jean‐Claude Chomel

**Affiliations:** ^1^ CHU de Poitiers, Service d’Oncologie Hématologique et Thérapie Cellulaire Poitiers France; ^2^ CHU de Poitiers, Service de Cancérologie Biologique Poitiers France; ^3^ CHU de Poitiers, Service d’Hématologie Biologique Poitiers France; ^4^ INSERM, CIC‐P Poitiers France

## Abstract

A large variety of molecular rearrangements of the *NUP98* gene have been described in the past decades (n = 72), involving fusion partners coding for different transcription factors, chromatin modifying enzymes, as well as various cytosolic proteins. Here, we report the case of an AML‐M2 patient with a variant *NUP98‐LEDGF/PSIP1* gene fusion (N9‐L10). In this patient, three different* NUP98-LEDGF* fusion mRNAs were characterized due to alternative splicing in *LEDGF* exon 11. Targeted high‐throughput sequencing revealed the presence of *IDH1*, *SRSF2*, and *WT1* additional pathogenic mutations. To improve the therapeutic monitoring, quantification of *NUP98*‐*LEDGF* mRNA by real‐time PCR was developed. Because of poor response to conventional chemotherapy, allogeneic stem cell transplantation was performed, followed by 20 cycles of azacitidine‐based preemptive treatment of relapse. More than 31 months after diagnosis, corresponding to 25 months post SCT and 4 months after the last cycle of azacytidine, the patient is in complete molecular remission (undetectable *NUP98‐LEDGF* mRNA transcripts). This study highlights the considerable variability in breakpoint location within both *NUP98* and *LEDGF*, associated with alternative splicing affecting *LEDGF*. It also emphasizes the need to fully characterize the breakpoints within the two genes and the identification of all fusion mRNAs, particularly for the development of a molecular monitoring assay. All these data seem critical for the optimal management of *NUP98*‐*LEDGF *+ hematological malignancies commonly associated with a poor prognosis.

## 1 INTRODUCTION

*NUP98* molecular rearrangements have been detected in acute myeloid leukemia (AML M1, M2, M4, M5, M7, and therapy‐related AML) and to a lesser extent in myelodysplastic syndromes (MDS, therapy‐related MDS) such as blast crisis‐chronic myeloid leukemia (BC‐CML), chronic myelomonocytic leukemia, juvenile myelomonocytic leukemia, and T‐cell acute lymphocytic leukemia.^1^, ^2^, ^3^ At least 72 *NUP98* fusion partner genes have been reported so far in the literature (http://atlasgeneticsoncology.org/Genes/GC_NUP98.html). The *NUP98* gene, located on chromosome 11p15.5, encodes a 98kDa nucleoporin component of the nuclear pore complex, which is a selective bidirectional nucleocytoplasmic transporter for macromolecules such as proteins, mRNA, tRNA, and ribosome subunits.^4^ It was recently shown that NUP98 fusion proteins could interact with MLL1 (mixed lineage leukemia 1) and NSL (nonspecific lethal) histone‐modifiers within a complex that binds HoxA and HoxB promoter regions and drives leukemogenic transformation *via* the enhanced expression of HOX genes.^5^

*LEDGF* (Lens Epithelium‐derived Growth Factor) gene on 9p22, also named *PSIP1* (PC4 and SFRS1 interacting protein 1) encodes two transcriptional coactivators (p75 and p52), generated by alternative splicing.^6^ LEDGF/p75 and LEDGF/p52 proteins, localized in the nucleus, are known to bind chromatin. LEDGF/p75 interacts with HIV‐1 integrase and promotes viral integration,^7^ while LEDGF/p52 (lacking the C‐terminal integrase‐binding domain of LEDGF/p75) can play a role in the regulation of splicing.^8^ Furthermore, it has been shown that LEDGF/p75 and MENIN1 bind to the N‐terminus of MLL protein. This trimeric complex is quite important, because it targets MLL and MLL‐fusion proteins to target gene promoters. In this respect, knockout mouse experiments have demonstrated that LEDGF/p75 has a critical role in the initiation of MLL‐rearranged leukemia.^9^

Rare t(9;11)(p22;p15) translocation with *NUP98*‐*LEDGF* (*NUP98*‐*PSIP1*) fusion has been described in adult patients with acute myeloid leukemia (M1/M2 AML),^10^, ^11^, ^12^, ^13^ transformed chronic myeloid leukemia,^14^ or myelodysplastic syndrome with excess blasts (MDS‐EB‐2),^15^ and in a pediatric case of transitional M2‐M3 AML.^16^

In this work, we have identified a novel *NUP98*‐*LEDGF* rearrangement in acute myeloid leukemia (AML‐M2). A comprehensive molecular analysis was conducted and three *NUP98‐LEDGF* fusion transcripts were fully characterized. Using these data, a specific qRT‐PCR assay was designed to evaluate the efficacy of the treatment. In addition to *NUP98*‐*LEDGF* rearrangement, high‐throughput sequencing has characterized additional *IDH1*, *SRSF2*, and *WT1* pathogenic variants. Because of the nature of *NUP98‐*related leukemia, the presence of additional genetic risk factors, and the absence of molecular remission after intensive induction chemotherapy, an allogeneic stem cell transplantation (SCT) was considered. Two and a half years after diagnosis and more than 2 years after SCT, the patient is still alive. He achieved complete remission without detectable minimal residual disease.

## 2 MATERIALS AND METHODS

### 2.1 Patient and IRB approval

The patient was admitted to our institution on February 2016, and the diagnosis of acute myeloid leukemia was made. He was included in a multicenter AML clinical trial (EudraCT Number: 2014‐000699‐24) and provided written informed consent in accordance with the declaration of Helsinki. For personal reasons, the patient withdrew from the trial 6 months later.

### 2.2 Flow cytometry

Flow cytometric immunophenotyping experiments were performed using CD2, CD3, CD5, CD7, CD10, CD11b, CD13, CD14, CD19, CD20, CD33, CD34, CD36, CD38, CD41b, CD56, CD61, CD64, CD71, CD117, cMPO, and HLA‐DR antibodies purchased from Beckman Coulter (Brea, CA), or Becton Dickinson (Franklin Lakes, NJ). Acquisition was carried out on a Navios 10‐color flow cytometer (Beckman Coulter), and blasts were identified according to the expression of myeloid, B lymphoid, T lymphoid, monocytic, erythroid, and megakaryocytic markers.

### 2.3 Cytogenetic analyses

Whole chromosome painting FISH (fluorescent in situ hybridization) was performed using the XCP 9 green and XCP 11 orange DNA FISH probes (MetaSystems, Altlussheim, Germany) following the manufacturer's instructions. Conventional cytogenetic and FISH images were analyzed by means of the IKAROS and ISIS software, respectively (MetaSystems).

### 2.4 Molecular experiments at diagnosis

Total RNA was extracted from both blood and bone marrow samples, and RT‐PCR experiments were performed using the N988F forward primer (within *NUP98 *exon 8) associated with p75R (within *LEDGF *exon 12) or p52R (within *LEDGF* exon 11b) reverse primer as previously described.^12^ Amplified fragments corresponding to *NUP98‐LEDGF* rearrangements were then purified from agarose gel by the QIAquick gel extraction kit (Qiagen, Hilden, Germany) and sequenced using the BigDye Terminator v3.1 Cycle Sequencing kit (Life Technologies, Austin, TX). For Next Generation Sequencing (NGS) experiments, genomic DNA was extracted from bone marrow, and the amplicon library was generated using the TruSight myeloid sequencing panel (Illumina, San Diego, CA) that targets most genes mutated in AML. Paired‐end sequencing was carried out on an Illumina MiSeq and data were analyzed by in‐house bioinformatics pipeline.

### 2.5 Molecular monitoring

To evaluate the molecular residual disease (MRD), the quantification of *NUP98‐LEDGF* fusion mRNA transcripts by real‐time RT‐PCR (qRT‐PCR) was implemented. A set of primers and probes was designed to amplify both *NUP98‐LEDGF* mRNAs detected in our study (forward primer, 5′‐GGCAGACCAATACTGGATTTG‐3′; reverse primer, 5′‐TCCTTCTGTGAGCAGTCTGAAAGT‐3′; TaqMan probe, 5′‐FAM‐CTGTTGGTTCGAAGAGAAAAGGTGGGAGG‐TAMRA‐3′). In these experiments, *ABL1 *was used as an internal control,^17^ and the kinetics of *NUP98‐LEDGF* fusion mRNA transcripts was assessed by the *NUP98‐LEDGF/ABL1* ratio. For each sample, *NUP98‐LEDGF* and *ABL1* mRNA quantifications were done in triplicate.

## 3 RESULTS

### 3.1 Case report

**Figure 1:**
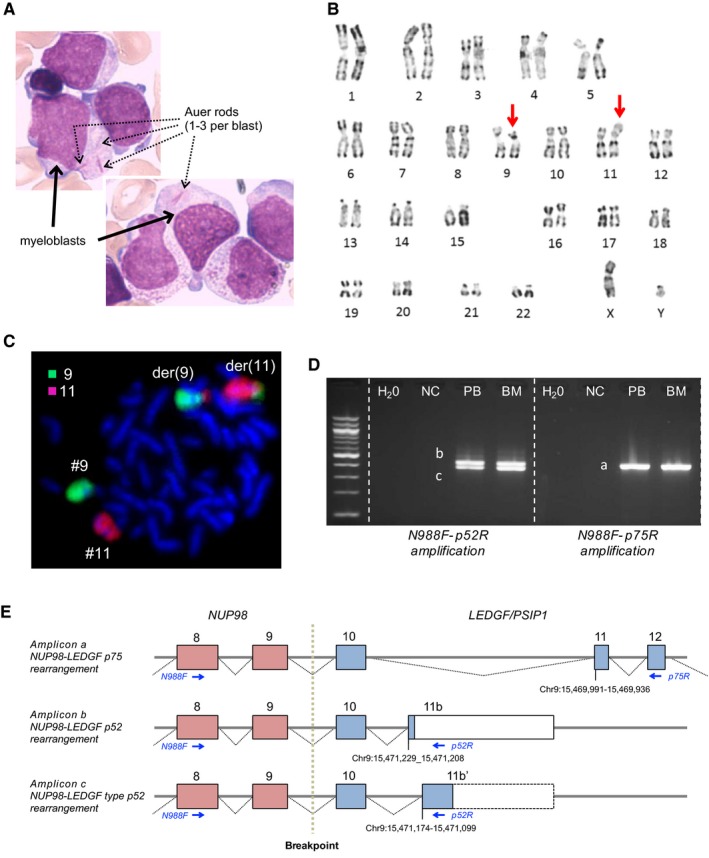
Cytological, cytogenetic, and molecular characteristics. (A) Bone marrow aspirate (May Grünwald Giemsa staining; magnification × 1000) revealed the presence of myeloblasts with isolated Auer rods (1‐3/blast cells). (B) Karyotype from bone marrow metaphases identified the t(9;11)(p22;p15) translocation. (C) Whole chromosome painting confirmed the presence of the t(9;11) translocation. (D) Three different *NUP98*‐*LEDGF* mRNA transcripts were detected by RT‐PCR. NC, negative control; PB, peripheral blood; BM, bone marrow. (D) Schematic representation of the three *NUP98‐LEDGF *rearrangements detected by RT‐PCR. Breakpoint between *NUP98* and *LEDGF* is shown, as well as the different isoforms due to *LEDGF* exon 11 alternative splicing. Genomic coordinates (GRCh37) of alternative exons 11 and location of primers are shown

A 58‐year‐old Caucasian patient presented with pancytopenia (hemoglobin 4.8 g/dL, white cell count 1 × 10^9^/L, neutrophils 0.1 × 10^9^/L, platelets 13 × 10^9^/L) and 76% circulating blasts. A bone marrow smear revealed moderate dyserythropoiesis and dysgranulopoiesis, and 70% myeloblasts (often with many Auer rods), suggesting AML with maturation (AML‐M2 according to the FAB classification) (Figure 1A). Flow cytometry confirmed the myeloid nature of blast cells that were positive for cMPO, CD13, CD33, CD38, and CD117 antigens, weakly positive for HLA‐DR, CD7, and CD34 antigens, and negative for lymphoid, monocytic, erythroid, and megakaryocytic markers (CD2, CD3, CD5, CD10, CD11b, CD14, CD19, CD20, CD36, CD41b, CD56, CD61, CD64, CD71). Cytogenetic analyses revealed a 46,XY,t(9;11)(p22;p15)[35]/46,XY[2] karyotype (Figure 1B). Whole chromosome painting confirmed the presence of the t(9;11)(p22;p15) reciprocal translocation (Figure 1C).

### 3.2 Characterization of the molecular rearrangement

Based on cytogenetic data, RT‐PCR experiments were performed on blood and bone marrow samples using the N988F forward primer associated with p75R or p52R reverse primer. Following agarose gel electrophoresis, three different amplicons corresponding to one *NUP98‐LEDGF*‐p75 fusion transcript and two *NUP98‐LEDGF*‐p52 fusion transcripts were observed (Figure 1D). These amplified fragments were recovered from agarose gel and sequenced. In all cases, *NUP98* exon 9 (N9) was fused in‐frame with *LEDGF* exon 10 (L10), and the three *NUP98‐LEDGF* fusion transcripts differed from an alternative exon 11 splicing within the *LEDGF* gene (Figure 1E).

More specifically, two splice isoforms corresponded to the usual *LEDGF*‐p75 *and LEDGF*‐p52 transcripts (NM_033222 and NM_001317900, respectively, Figure S1A,B). The third isoform was related to an unknown variant of *LEDGF*‐p52 due to the presence of an unusual exon 11 (Figure S1C). Using the Human Splicing Finder (www.umd.be/HSF3/) and Sroogle (http://sroogle.tau.ac.il) software, one splice acceptor site and several branch points were predicted upstream this atypical *LEDGF* exon 11 (Figure S2). The potential impact of the detection in the blood and bone marrow samples from our patient of three *NUP98*‐*LEDGF* fusion transcripts differing due to an alternative *LEDGF* exon 11, including one that was unknown, remains to be determined. Moreover, the two shorter versions of NUP98‐LEDGF protein lack the IBD domain which would connect these fusion proteins to MLL biology (Figure S3). Overall, we report a novel N9/L10 *NUP98*‐*LEDGF* molecular rearrangement distinct from N8/L3,^12^ N9/L5,^16^ N9/L7,^11^, ^14^ N11/L8,^15^ and N12/L8^15^ junctions previously reported in the literature (Table S1 and Figure S3).

### 3.3 Targeted NGS analysis

Genomic DNA from bone marrow at diagnosis was then analyzed by NGS on a MiSeq sequencer using the TruSight Myeloid sequencing panel. Three genetic variants were characterized: *IDH1* p.Arg132His, *SRSF2* p.Pro95Thr, and *WT1* p.Cys393Ter (Table S2). *WT1 *(tumor suppressor), *SRSF2* (a component of the spliceosome machinery), and *IDH1* (involved in DNA methylation) were found to be recurrently mutated in AML patients.^18^ The pathogenic mutations of *IDH1* (codon 132) and *SRSF2* (codon 95) are commonly involved in MDS, AML associated with MDS and de novo AML. In the present case, *IDH1* p.Arg132His and *SRSF2* p.Pro95Thr mutations were detected at high variant allele frequency (VAF > 40%). The *WT1* p.Cys393Ter variant (VAF 7%) has not yet been reported but appears to be probably pathogenic since it introduces a stop codon in the WT1 tumor suppressor protein.

### 3.4 Treatment and molecular follow‐up

**Figure 2:**
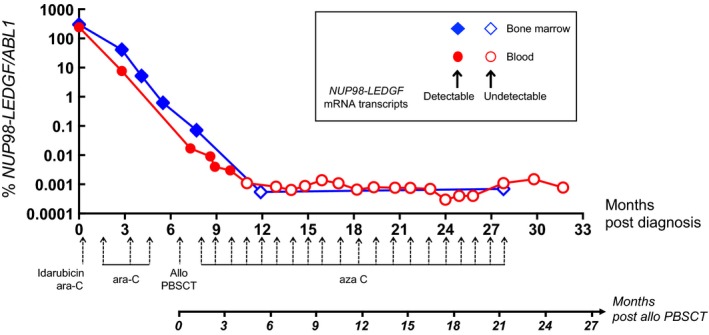
Molecular monitoring. Follow‐up of the molecular disease by the quantification of *NUP98*‐*LEDGF* mRNA transcripts in blood and bone marrow samples from the patient reported here. The *x*‐axis represents the duration of the molecular monitoring (in months). Consecutive treatments are detailed below the curve. Ara‐C, cytarabine; PBSCT, peripheral blood stem cell transplantation; aza C, azacitidine. Full circles and squares represent *NUP98‐LEDGF *positive samples assessed by the *NUP98‐LEDGF/ABL1* ratio. Open circles and squares represent *NUP98‐LEDGF* negative samples assessed by sensitivity of the test (1/number *ABL1 *copies)

Standard intensive induction chemotherapy treatment (idarubicin 9 mg/m^2^, days 1‐5 and cytarabine 200 mg/m^2^, days 1‐7) failed to induce a complete hematologic remission (46% bone marrow blast cells at day 44 with persistent aplasia). Complete remission with incomplete hematological recovery (CRi) was subsequently achieved using a cytarabine‐based salvage treatment (1500 mg/m^2^/12 h, day 1, 3, 5). However, *NUP98‐LEDGF* mRNA transcripts remained detectable, in both peripheral blood and bone marrow (Figure 2). Two consolidation cycles were then carried out with the same treatment protocol allowing CRi to be maintained. In this context, an allogeneic SCT was performed with an unrelated HLA‐matched (10/10) donor. Peripheral blood stem cells were injected after a reduced intensity conditioning (fludarabine 30 mg/m^2^ on days −6 to −2; busulfan 3.2 mg/kg on days −4 to −3 and thymoglobulin 5 mg/kg on days −3 to −2). Cyclosporine and mycophenolate mofetil were used as prophylaxis against Graft versus Host Disease (GvHD). The procedure was first complicated with acute cutaneous and gastrointestinal GvHD, and then successfully treated with corticosteroids. At 7 months posttransplantation, the patient developed a moderate chronic mucocutaneous GvHD that resolved with corticosteroid and rapamycin therapy. Identified as a patient at high risk of relapse, it was decided that he would receive azacitidine as preemptive treatment (75 mg/m^2^/day for five consecutive days every 28 days) beginning day 48 posttransplant. Concerning molecular monitoring, the *NUP98‐LEDGF*/*ABL1* ratio continuously decreased from the diagnosis to approximately 5 months posttransplant (Figure 2). At this point, *NUP98‐LEDGF* transcripts became undetectable, in both peripheral blood and bone marrow. Starting 5 months post allotransplant and up until now (more than 25 months post SCT and around 4 months after the last cycle of azacitidine), *NUP98‐LEDGF* fusion transcripts have remained undetectable in peripheral blood (*ABL1*>60 000 copies in each experiment performed in triplicate). In addition, qRT‐PCR performed at the time of the 20th and last cycle of azacitidine was negative in blood as well as in bone marrow samples. Overall, the patient achieved CR without minimal residual disease (CR_MRD‐_).

## 4 DISCUSSION

Very few cases of *NUP98‐LEDGF*+hematological malignancies have been reported.^10^, ^11^, ^12^, ^13^, ^14^, ^15^, ^16^ At diagnosis, the median age of patients was 52 (range 5‐64) years and the median WBC was 50.5 (range 1.5‐293) × 10^9^/L. However, at presentation, three patients had pancytopenia and two showed hyperleukocytosis. The median survival time was 8.3 (range 3‐54) months. Overall, despite heterogeneous clinical and biological features, *NUP98‐LEDGF*+ hematological malignancies usually present as very aggressive disorders.

As shown by previously published data and by us in the present study, the high variability in breakpoint locations within both *NUP98* (4) and *LEDGF *(5) is a peculiar characteristic of *NUP98‐LEDGF* fusions.^11^, ^12^, ^14^, ^15^, ^16^ In all cases, *NUP98‐LEDGF*‐p75 and *NUP98‐LEDGF*‐p52 fusion transcripts arising via alternative splicing within *LEDGF* were detected. In addition to the present work, another group identified additional *NUP98‐LEDGF*‐type p52 fusions characterized by unusual splicing in *LEDGF*.^15^ Therefore, *NUP98‐LEDGF* fusions mostly differ from one patient to another. Theoretically, these rearrangements should encode fusion proteins harboring different structures and consequently displaying distinct functions. Theoretically, the *NUP98‐LEDGF *fusions characterized in this work should encode three NUP98‐LEDGF proteins (p75, p52, and type p52 variants). However, only variant p75 contains LEDGF integrase‐binding domain which can interact with MLL to form the trimeric complex with MENIN1. This suggests that only the largest NUP98‐LEDGF fusion protein is presumably able to bind to wild type MLL. However, from a prognostic point of view, the type of molecular rearrangement does not seem to correlate with clinical course. Nevertheless, complete characterization of breakpoints within *NUP98* and *LEDGF* and identification of all fusion mRNA transcripts seem mandatory, particularly for implementation of a molecular monitoring system based on qRT‐PCR.

Targeted NGS analysis performed at diagnosis and focused on acute myeloid leukemia driver genes is helpful since the presence of pathogenic mutants (*IDH1* p.Arg132His, *SRSF2* p.Pro95Thr, *WT1* p.Cys393Ter in this study) could represent additional risk factors or opportunities for targeted therapy. Indeed, *IDH1* mutations have an adverse prognostic effect in AML and constitute a drug‐targetable gene alteration.^19^, ^20^ *SRSF2* mutations are classically associated with poor outcome.^21^, ^22^ *WT1* mutations are defined as a secondary event in AML and the unknown nonsense variant described here could have a pejorative impact.^23^

In conclusion, we performed a comprehensive molecular analysis of a *NUP98‐LEDGF* fusion detected in the blood and bone marrow samples from a patient with AML. A novel molecular rearrangement was characterized giving rise to three fusion mRNA transcripts. In addition, myeloid‐focused NGS was performed, and a specific qRT‐PCR system was developed for molecular follow‐up. At the clinical level, an allogeneic SCT was performed because of (i) a rearrangement known to cause aggressive hemopathies, (ii) detection of additional *IDH1*, *SRSF2*, and *WT1 *point mutations, and (iii) the absence of molecular remission after intensive induction chemotherapy. More than 31 months after diagnosis, the patient achieved complete molecular remission. All in all, complete genetic characterization of the disease and implementation of personalized *NUP98‐LEDGF* mRNA quantification facilitated therapeutic decisions and monitoring of treatment efficacy. More generally, in the case of *NUP98‐LEDGF* fusion, allogeneic SCT should be considered as a reasonable therapeutic option, depending on the age of the patient, genetic factors and response to first‐line conventional chemotherapy. In this regard, molecular monitoring based on the quantification of *NUP98‐LEDGF* mRNA transcripts first in bone marrow (until the achievement of molecular remission) and then in peripheral blood appears critical for the evaluation of residual disease in malignancies classically associated with a poor prognosis.
