# Supplementary material for: Long‐term molecular remission in a patient with acute myeloid leukemia harboring a new NUP98‐LEDGF rearrangement
Source: Cancer Med. 2019 Mar 7;8(4):1765–70. doi: 10.1002/cam4.2051 (PMC6488106; doi:10.1002/cam4.2051)

SUPPORTING INFORMATION

Supporting Information Table S1: Main characteristics of patients with a t(9;11)(p22;p15) translocation.

Supporting Information Table S2: Mutations detected by targeted NGS analysis.

Supporting Information Figure S1: Protein and nucleotide sequences of the three N9/L10 *NUP98-LEDGF* fusion mRNA transcripts detected in the present study. In all cases, *NUP98* exon 9 is fused with *LEDGF* exon 10. A) Fusion with *LEDGF*-p75. B) Fusion with *LEDGF*-p52. C) Fusion with unknown *LEDGF*-type p52. Open triangles distinguish exons and solid triangles represent the breakpoint location.

Supporting Information Figure S2: *LEDGF* alternative exon 11 in novel N9/L10 *NUP98-LEDGF* mRNA transcripts detected in the present study. Location of splice donor/acceptor sites and potential branch points is shown. The prediction of potential splicing sites in *LEDGF* sequences was performed using Human Splicing Finder and Sroogle software online.

Supporting Information Figure S3: Breakpoint location heterogeneity in *NUP98-LEDGF* rearrangements. The positions of known breakpoints within *NUP98* and *LEDGF* genes are indicated. Breakpoints that cause *NUP98-LEDGF* gene fusions characterized in the present work are highlighted (N9-L10), as well as the three potential fusion proteins resulting from alternative splicing. The different functional domains of the two proteins are also shown. For NUP98; FG: phenylalanine-glycine, GLEBS: gle2p-binding-like motif, the nucleoporin 2 domain contains a RNA-binding motif. For LEDGF; PWWP, Pro-Trp-Trp-Pro Domain; CR, Charged Region; NLS, Nuclear localization signal; AT, AT-Hook; IBD, Integrase-Binding Domain.

Supporting Information Table S1: Main characteristics of patients with a t(9;11)(p22;p15) translocation.

Reference	Sex	Age (Years)	WBC (10^9/L)	Diagnosis	Survival (months)	*NUP98-LEDGF* fusion	Other abnormalities
Ha et al. 1994	F	20	63.8	AML M1	3		
Ahuja et al. 2000	M	52	50,5	AML M1	9	N9/L7	
Hussey et al. 2001	F	60	1,5	AML M2	54	N8/L3	
Grand et al. 2005	M	29	293	BC-CML	1.5	N9/L7	
Morerio et al. 2005	F	5	207	AML M2-M3		N9/L5	
Lundin et al. 2011	F	64	2.5	AML M2	24		FLT3-ITD
Yamamoto et al. 2012	F	64	1.8	MDS-EB-2	7.5	N11/L8<br>N12/L8	
This report	M	58	1.0	AML M2	> 31	N9/L10	IDH1, SRSF2, WT1 mutations in NGS

Supporting Information Table S2: Mutations detected by targeted NGS analysis.

**Gene transcript reference**	NM_005896	NM_003016	NM_024426
**HGNC gene symbol**	***IDH1***	***SRSF2***	***WT1***
**Genomic coordinates (GRCh37)**	2:209113112	17:74732960	11:32417868…32417883
**CDS variant**	c.395G>A	c.283C>A	c.1179_1194del16insA
**AA variant**	p.Arg132His	p.Pro95Thr	p.Cys393Ter
**VAF**	0.45	0.53	0.07
**Pathogenicity**	Pathogenic	Pathogenic	Probably pathogenic (nonsense mutation)
	AML<br>AML associated with MDS<br>MDS	MDS<br>CMML<br>AML<br>AML associated with MDS	Unknown

AML, acute myeloid leukemia; MDS, myelodysplastic syndrome; CMML, chronic myelomonocytic leukemia.

Supporting Information Figure S1: Protein and nucleotide sequences of the three *NUP98-LEDGF* fusion mRNA transcripts detected in the present study.

A – *NUP98-LEDGF*-p75 (amplicon a)

```
 G  N  T  S  T  I  G  Q  P  S  T  N  T  M  G  L  F  G  V  T
TTGGTAATACCAGCACCATAGGACAGCCAAGCACCAACACCATGGGATTATTTGGAGTAA
          NUP98 exon 8                     △
 Q  A  S  Q  P  G  G  L  F  G  T  A  T  N  T  S  T  G  T  A
CCCAAGCCTCACAGCCTGGAGGTCTTTTTGGGACAGCTACAAACACCAGCACTGGGACAG

 F  G  T  G  T  G  L  F  G  Q  T  N  T  G  F  G  A  V  G  S
CATTTGGAACAGGAACAGGTCTCTTTGGGCAGACCAATACTGGATTTGGTGCTGTTGGTT
           NUP98 exon 9
 K  R  K  G  G  R  N  F  Q  T  A  H  R  R  N  M  L  K  G  Q
CGAAGAGAAAAGGTGGGAGGAACTTTCAGACTGCTCACAGAAGGAATATGCTGAAAGGCC
 ▲                                  LEDGF/PSIP1 exon 10
 H  E  K  E  A  A  D  R  K  R  K  Q  E  E  Q  M  E  T  E  Q
AACATGAGAAAGAAGCAGCAGATCGAAAACGCAAGCAAGAGGAACAAATGGAAACTGAGC

 Q  N  K  D  E  G  K  K  P  E  V  K  K  V  E  K  K  R  E  T
AGCAGAATAAAGATGAAGGAAAGAAGCCAGAAGTTAAGAAAGTGGAGAAGAAGCGAGAAA
△                 LEDGF/PSIP1 exon 11                    △
 S  M  D  S  R  L  Q  R  I  H  A  E  -  -  -  -  -  -
CATCAATGGATTCTCGACTTCAAAGGATACATGCTGAG---------------
     LEDGF/PSIP1 exon 12
```

LEDGF/PSIP1
NM_033222
splicing variant
(p75 isoform)

B - *NUP98-LEDGF*-p52 (amplicon b)

```
 G  N  T  S  T  I  G  Q  P  S  T  N  T  M  G  L  F  G  V  T
TTGGTAATACCAGCACCATAGGACAGCCAAGCACCAACACCATGGGATTATTTGGAGTAA
          NUP98 exon 8                     △
 Q  A  S  Q  P  G  G  L  F  G  T  A  T  N  T  S  T  G  T  A
CCCAAGCCTCACAGCCTGGAGGTCTTTTTGGGACAGCTACAAACACCAGCACTGGGACAG

 F  G  T  G  T  G  L  F  G  Q  T  N  T  G  F  G  A  V  G  S
CATTTGGAACAGGAACAGGTCTCTTTGGGCAGACCAATACTGGATTTGGTGCTGTTGGTT
           NUP98 exon 9
 K  R  K  G  G  R  N  F  Q  T  A  H  R  R  N  M  L  K  G  Q
CGAAGAGAAAAGGTGGGAGGAACTTTCAGACTGCTCACAGAAGGAATATGCTGAAAGGCC
 ▲                                  LEDGF/PSIP1 exon 10
 H  E  K  E  A  A  D  R  K  R  K  Q  E  E  Q  M  E  T  E  H
AACATGAGAAAGAAGCAGCAGATCGAAAACGCAAGCAAGAGGAACAAATGGAAACTGAGC

 Q  T  T  C  N  L  Q  *
ACCAAACAACATGTAATCTACAGTAA
△   LEDGF/PSIP1 exon 11b
```

LEDGF/PSIP1
NM_001314900
splicing variant
(p52 isoform)

C - *NUP98-LEDGF*-type p52 (amplicon c)

```
 G  N  T  S  T  I  G  Q  P  S  T  N  T  M  G  L  F  G  V  T
TTGGTAATACCAGCACCATAGGACAGCCAAGCACCAACACCATGGGATTATTTGGAGTAA
          NUP98 exon 8                     △
 Q  A  S  Q  P  G  G  L  F  G  T  A  T  N  T  S  T  G  T  A
CCCAAGCCTCACAGCCTGGAGGTCTTTTTGGGACAGCTACAAACACCAGCACTGGGACAG

 F  G  T  G  T  G  L  F  G  Q  T  N  T  G  F  G  A  V  G  S
CATTTGGAACAGGAACAGGTCTCTTTGGGCAGACCAATACTGGATTTGGTGCTGTTGGTT
           NUP98 exon 9
 K  R  K  G  G  R  N  F  Q  T  A  H  R  R  N  M  L  K  G  Q
CGAAGAGAAAAGGTGGGAGGAACTTTCAGACTGCTCACAGAAGGAATATGCTGAAAGGCC
 ▲                                  LEDGF/PSIP1 exon 10
 H  E  K  E  A  A  D  R  K  R  K  Q  E  E  Q  M  E  T  E  H
AACATGAGAAAGAAGCAGCAGATCGAAAACGCAAGCAAGAGGAACAAATGGAAACTGAGC

 I  N  P  V  T  E  K  R  I  Q  V  E  Q  T  R  D  E  D  L  D
ACATTAATCCAGTTACTGAAAAGAGAATACAAGTGGAGCAAACAAGAGATGAAGATCTTG
△                                LEDGF/PSIP1 exon 11b'
 T  D  S  L  D  *
ATACAGACTCATTGGACTGA
```

LEDGF/PSIP1
unknown
splicing variant
(type 52 isoform)

Supporting Information Figure S2: *LEDGF* alternative exon 11 in novel N9/L10 *NUP98-LEDGF* mRNA transcripts detected in the present study.

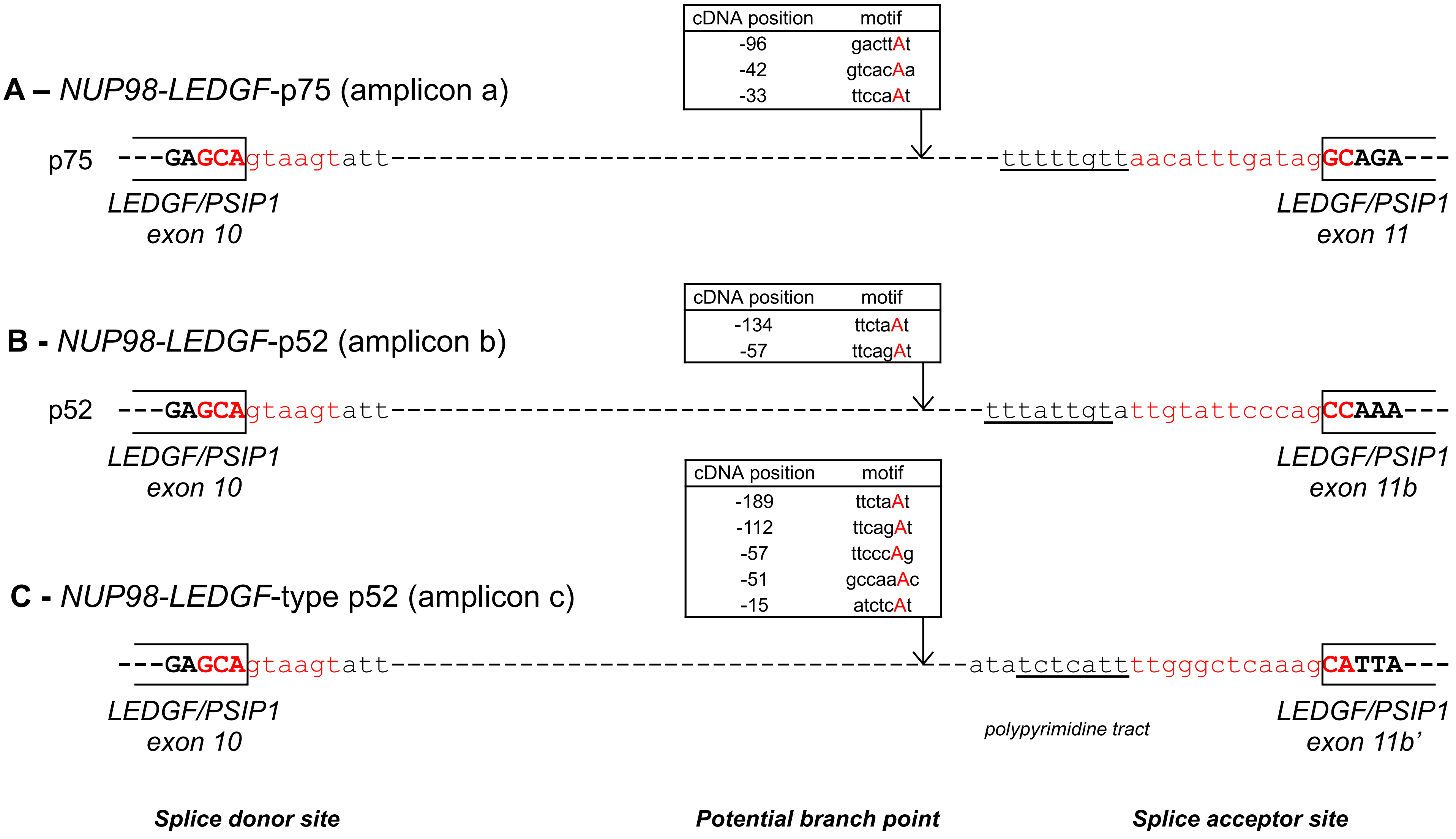

Supporting Information Figure S3: Fusion Breakpoint location heterogeneity in *NUP98-LEDGF* rearrangements.

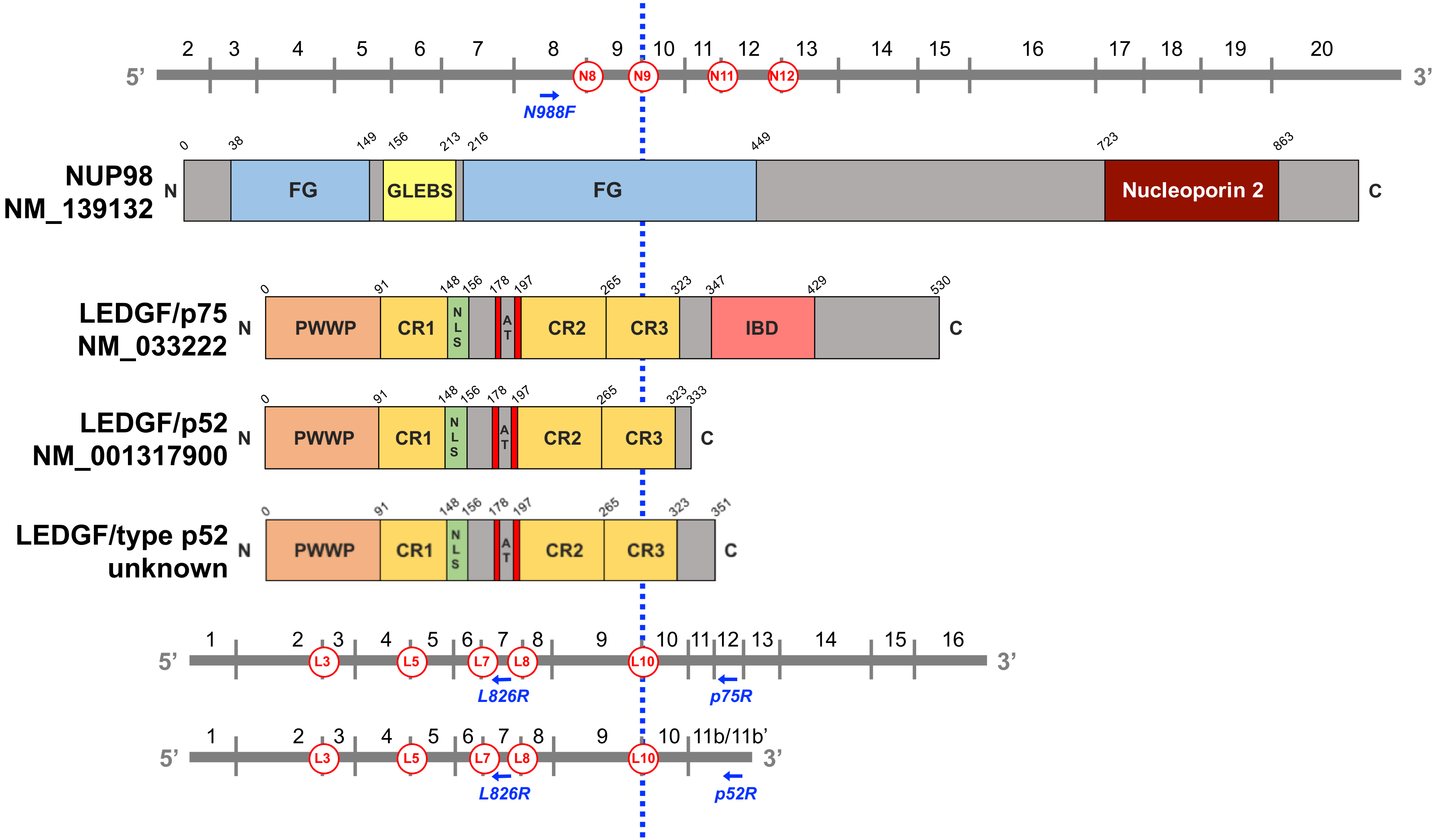

Supplement: Supplementary file 1 [file CAM4-8-1765-s001.pdf]
